# Indocyanine green fluorescence imaging for accurate detection of an intramural coronary artery during robotic coronary artery bypass grafting

**DOI:** 10.1016/j.xjtc.2025.01.018

**Published:** 2025-01-28

**Authors:** Zain Khalpey, Ujjawal Kumar, Zacharya Khalpey, Tyler Phillips, Feras Khaliel

**Affiliations:** a Department of Cardiac Surgery, HonorHealth, Scottsdale, Ariz; b Khalpey AI Lab, Applied & Translational AI Research Institute, Scottsdale, Ariz; c School of Clinical Medicine, University of Cambridge, Cambridge, United Kingdom; d Department of Cardiac Surgery, King Faisal Specialist Hospital and Research Center, Riyadh, Saudi Arabia

**Figure:**
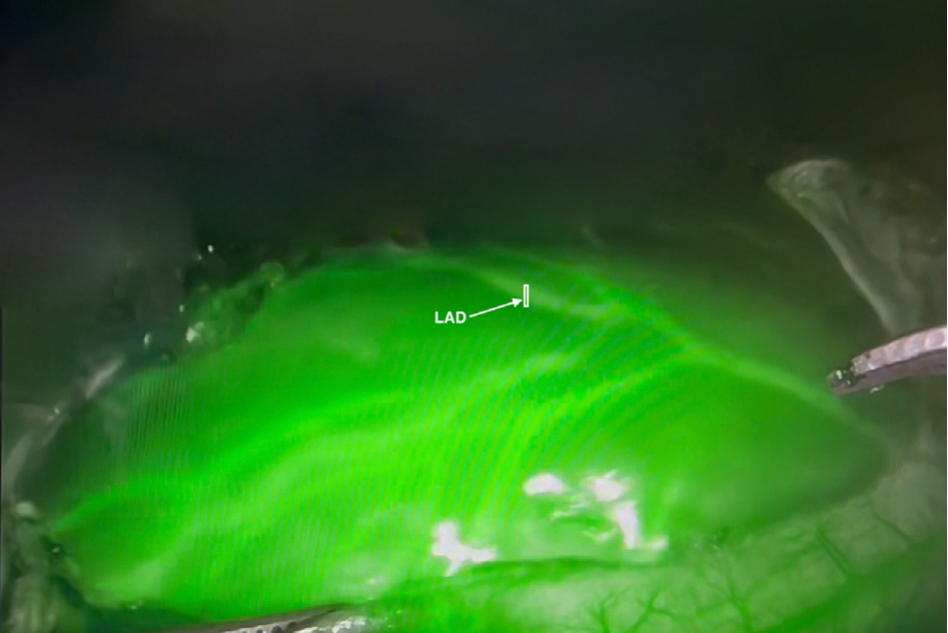
Successful intraoperative visualization of the LAD using ICG and Firefly mode.

**Central Message**

Intraoperative fluorescence imaging using indocyanine green enabled precise visualization of an intramural left anterior descending coronary artery during robotic MIDCAB, facilitating successful coronary revascularization.

Intramural coronary arteries provide technical challenges during coronary artery bypass grafting (CABG). Found in approximately 42% of individuals^1^ these vessels can lead to myocardial ischemia, arrhythmias, and sudden cardiac death. Traditional angiography has significant limitations in precisely localizing intramural segments,^2^ resulting in prolonged dissection/operative times and increasing the risk of myocardial damage. We present near-infrared fluorescence (NIRF) imaging as a solution for intraoperative visualization of intramural vessels. Institutional review board approval requirement waived; written patient consent for publication was received.

## Case Report

**Figure 1:**
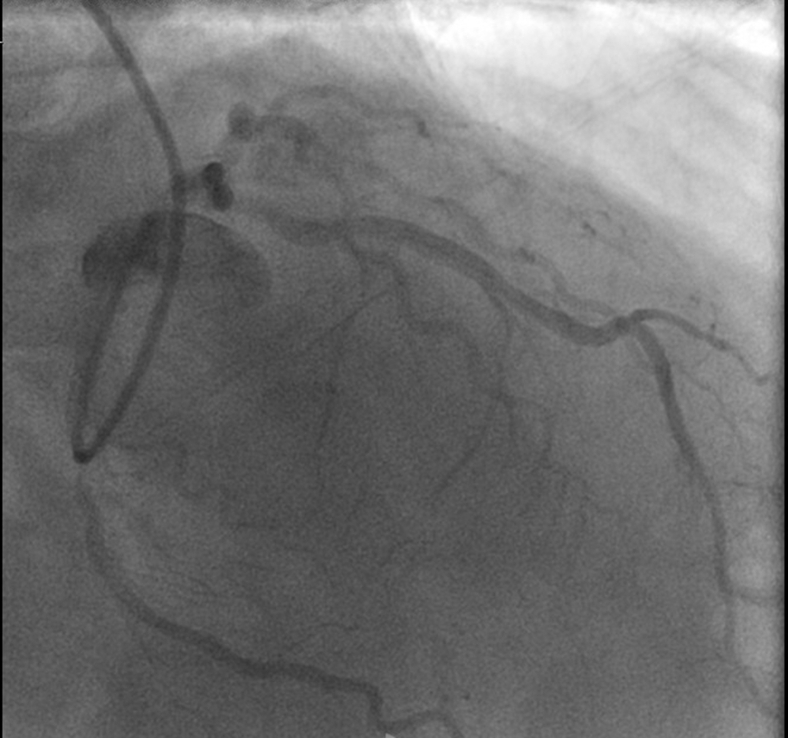
Preoperative angiography: proximal left anterior descending coronary artery and mid-left circumflex artery stenosis.

A 57-year-old man with a history of hyperlipidemia and prior orthopedic surgeries was evaluated. Preoperative cardiac catheterization (Figure 1) revealed 90% proximal left anterior descending artery (LAD) stenosis and 70% midvessel stenosis in the left circumflex artery (LCx), correlating with anterior and inferior wall ischemia on nuclear stress testing (left ventricular ejection fraction, 67%). An intramural course of the LAD was also noted. Because postoperative rehabilitation would be challenging (the patient has severely limited mobility), hybrid revascularization with robotic minimally invasive direct coronary artery bypass (MIDCAB) to the LAD and staged percutaneous coronary intervention to the LCx was planned.

**Figure 2:**
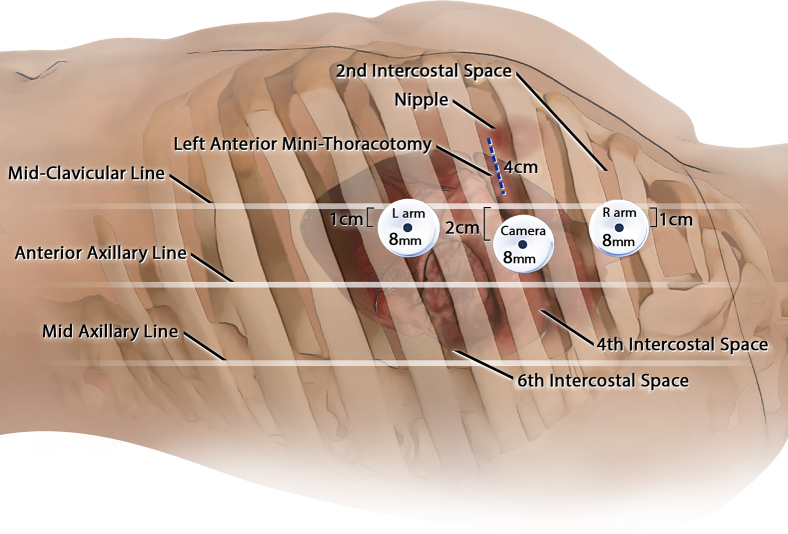
Setup for robot-assisted minimally invasive direct coronary artery bypass.

**Figure E1:**
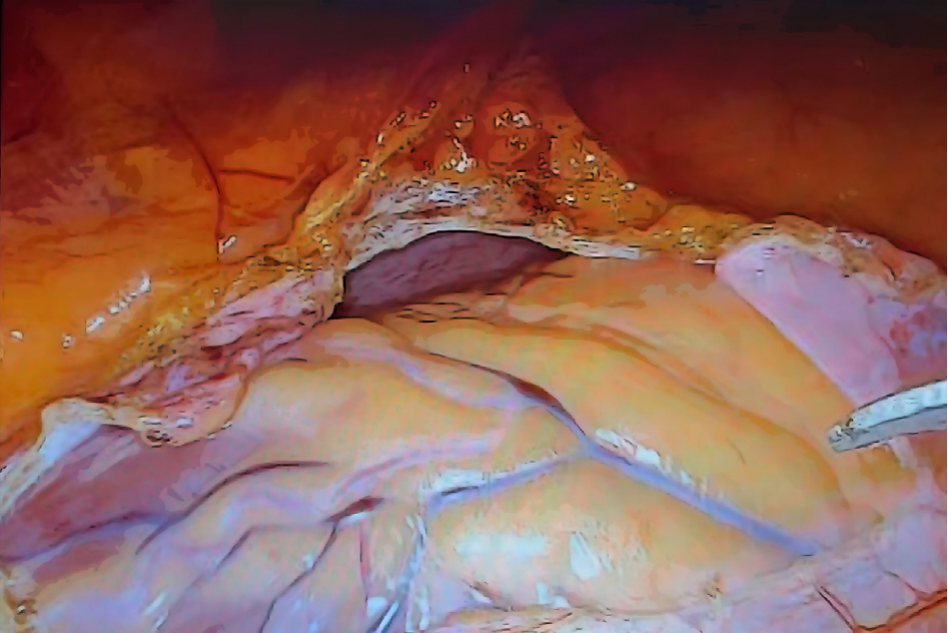
Left ventricular epicardial surface. The intramural left anterior descending coronary artery was difficult to visualize.

**Figure E2:**
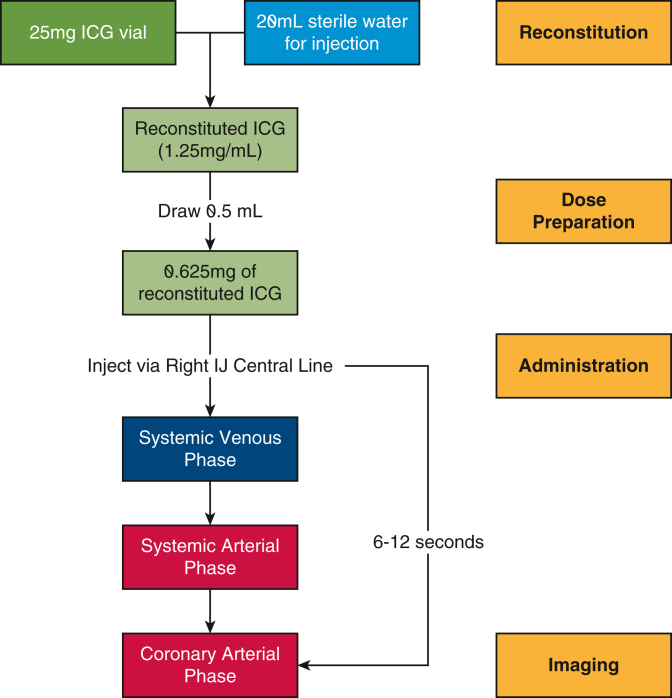
Protocol for indocyanine green (ICG) preparation and administration. *IJ*, Internal jugular.

**Figure E3:**
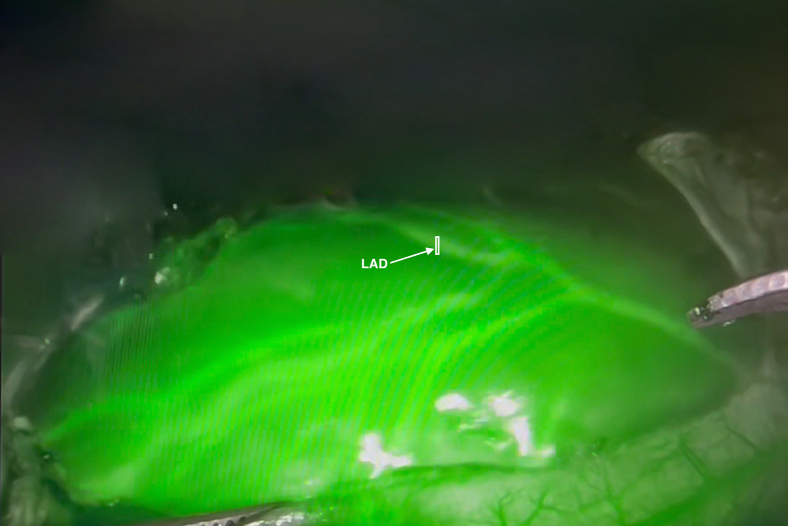
Fluorescence view following indocyanine green administration; the course of the left anterior descending coronary artery (LAD) is now visible. A mammary clip was placed on the overlying epicardial fat as a marker.

Incisions were made, ports were introduced, and the da Vinci Xi robotic system (Intuitive) was docked (Figure 2). The left internal thoracic artery was robotically harvested with a 1-cm intrathoracic fascial pedicle. Visualizing the LAD's course proved challenging (Figure E1). Indocyanine green (ICG) was prepared (Figure E2) and 0.5 mL of reconstituted ICG solution (1.25 mg/mL) was administered following pericardial opening. Fluorescence imaging (Firefly; Intuitive) in the arterial phase delineated the LAD's path (Figure E3), and a mammary clip was placed on the overlying epicardial fat as a marker allowing the anastomosis to be completed in the typical MIDCAB technique. Native flow was temporarily occluded with silastic tapes and the flow was measured using Medistim (Medistim), showing excellent graft function (pulsatility index, 2.1; flow, 12 mL/min). Intercostal nerves 3 through 5 were ablated for postoperative pain management using a cryoprobe (cryoICE; AtriCure). Echocardiography demonstrated preserved ejection fraction with negligible valvular insufficiency.

Postoperative recovery was uncomplicated; he was discharged from the intensive care unit on postoperative day 1 and from the hospital on postoperative day 3. At 2 weeks, he reported minimal discomfort managed with acetaminophen only and was walking 1 mile daily. Staged percutaneous coronary intervention to the left circumflex coronary artery completed revascularization 1 month postoperatively. The interventional cardiologist confirmed a patent graft to the LAD. At 9 months, the patient has had no further anginal episodes or electrocardiogram changes, with increasing exercise tolerance and no residual pain.

We present the successful application of robotic cardiac surgery in a patient with functional limitations that achieved complete revascularization while avoiding sternotomy. The use of ICG-guided NIRF imaging facilitated precise grafting and minimized operative time and the minimally invasive approach enabled rapid postoperative recovery.

## Comment

Successful identification and bypass of intramural coronary arteries represents a significant technical challenge in coronary revascularization. This case demonstrates the novel application of ICG-guided NIRF imaging to localize an intramural LAD during robotic MIDCAB, preventing conversion to sternotomy. Although ICG-guided NIRF imaging is well established for evaluating anastomoses and graft flow in CABG surgery,^3^ its utility in delineating intramural coronary anatomy is novel. Further, the literature is sparse regarding its use for identifying the course of intramural coronary vessels.

NIRF coronary angiography with ICG has shown promise in providing real-time visualization of coronary arteries, bypass grafts, and myocardial perfusion during CABG surgery.^4^ Developments in imaging systems have allowed simultaneous capture of color and NIR images, improving visualization of both grafts and perfusion abnormalities.^5^ ICG-NIRF imaging allows real-time visualization of coronary anatomy, reduced need for extensive dissection to identify vessel course, and assessment of graft patency at completion. In minimally invasive approaches such as totally endoscopic coronary artery bypass, it could ensure the correct trajectory is maintained during LAD dissection. NIRF could aid robotic grafting to the right coronary artery, addressing the challenge of vessel localization in minimally invasive approaches. This aligns with broader trends toward minimally invasive cardiac surgery and increasing integration of advanced imaging technologies in surgical decision making.

## Conflict of Interest Statement

The authors reported no conflicts of interest.

The *Journal* policy requires editors and reviewers to disclose conflicts of interest and to decline handling or reviewing manuscripts for which they may have a conflict of interest. The editors and reviewers of this article have no conflicts of interest.
